# Xenon prevents cellular damage in differentiated PC-12 cells exposed to hypoxia

**DOI:** 10.1186/1471-2202-5-55

**Published:** 2004-12-08

**Authors:** Christian Petzelt, Per Blom, Wolfgang Schmehl, Jana Müller, Wolfgang J Kox

**Affiliations:** 1 University Hospital Charité, Clinic for Anesthesiology and Intensive Care, Experimental Anesthesiology, 14050 Berlin, Germany; 2 Linde Gas Therapeutics, 18181-Lidingö, Sweden

## Abstract

**Background:**

The neuroprotective effect of xenon has been demonstrated for glutamatergic neurons. In the present study it is investigated if dopaminergic neurons, i.e. nerve-growth-factor differentiated PC-12 cells, are protected as well against hypoxia-induced cell damage in the presence of xenon.

**Results:**

Pheochromocytoma cells differentiated by addition of nerve growth factor were placed in a N_2_-saturated atmosphere, a treatment that induced release of dopamine, reaching a maximum after 30 min. By determining extracellular lactate dehydrogenase concentration as marker for concomitant cellular damage, a substantial increase of enzymatic activity was found for N_2_-treated cells. Replacement of N_2 _by xenon in such a hypoxic atmosphere resulted in complete protection against cellular damage and prevention of hypoxia-induced dopamine release. Intracellular buffering of Ca^2+ ^using the Ca-chelator 1, 2-*bis*(2-Aminophenoxy)ethane-N,N,N',N'-tetraacetic acid *tetrakis*(acetoxymethyl) ester (BAPTA) reduced the neuroprotective effect of xenon indicating the essential participation of intracellular Ca^2+^-ions in the process of xenon-induced neuroprotection.

**Conclusions:**

The results presented demonstrate the outstanding property of xenon to protect neuron-like cells in a hypoxic situation.

## Background

Originally, hypoxia/ischemia-induced alterations in neuronal function have been attributed to be an over-release of neurotransmitters, including dopamine and glutamate. Many studies have been performed on the mechanisms of glutamate-induced neuronal damage [1,2] but relatively few have investigated the hypoxia-induced damage in dopaminergic neurons [3-6]. In recent years several lines of evidence have suggested that effects other than excitotoxic mechanisms may also participate in hypoxia-induced cell damage such as cortical spreading depression [7,8]. Rat pheochromocytoma (PC-12) cells are catecholaminergic, excitable cells that have been widely used as an *in vitro *model for neuronal cells [9] possessing both D1- and D2-dopamine receptors [10]. In these cells hypoxia causes a transient release of dopamine resulting from a complex cellular response consisting of increased dopamine release and reduced uptake rate. Such increased dopamine concentration has been shown to be associated with cellular damage indicated by an elevated release of lactate dehydrogenase (LDH) from the cells [6,11].

Numerous approaches have been undertaken to reduce hypoxia-induced neurotoxicity [2,12]. The pathological increase of extracellular neurotransmitter concentration presents probably one of the first indicators for such damage although it is not clear to what extent it contributes directly. Thus, a reduction or even complete suppression of such an increase of neurotransmitter concentration after the primary neuronal damage would suggest a high probability for protection from the hypoxic insult. Recently, we have shown that the noble gas xenon prevents in hypoxic cortical neurons hypoxia-induced cell damage and glutamate release [13,14]. Such neuroprotective potential has been confirmed by Ma et al. [15] and Wilhelm et al.[16], and related to its property of being an NMDA-receptor antagonist. In the present paper, however, we show that also in the dopaminergic PC-12-system xenon exhibits profound neuroprotective properties for hypoxic cells thus underlining its usefulness as a general neuroprotectant.

## Results

### Release of dopamine under hypoxic conditions

**Figure 1:**
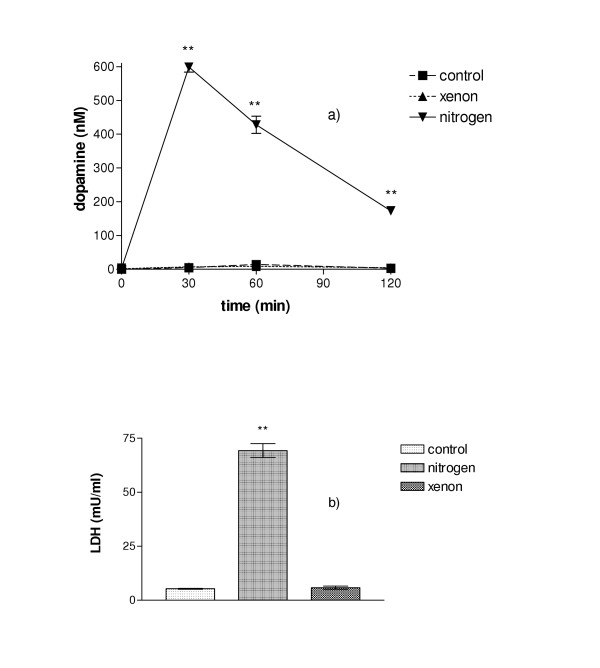
A. Dopamine release from differentiated PC-12 cells under normoxic conditions, in N_2_, or in xenon. Whereas almost no dopamine was released from control cells during the two-hour period, a strong increase of extracellular dopamine was found when cells were kept in N_2_. When cells were maintained in a xenon-atmosphere, no dopamine release occurred, there was virtually no difference compared to controls. B. Assessment of cellular damage in PC-12 cells after two-hour incubation. If cells were kept in normal air, or in xenon, only a small amount of LDH was released. Much higher cellular damage was found when cells were incubated in N_2_. (n = 5; **P < 0.01 with respect to untreated controls).

Cells kept under normoxic conditions did not release dopamine during the time period studied. If, however, they were kept in an atmosphere consisting of 100% nitrogene, considerable amounts of dopamine were found in the extracellular space reaching a maximum at 30 min of incubation, followed by a subsequent decrease. If under the same conditions nitrogen was replaced by xenon, no such increase in dopamine concentration was found (Fig. 1a). The level of extracellular dopamine remained as low as in cells kept under normoxic conditions.

### Hypoxia-induced cellular damage

In order to test if such hypoxia damaged the cells, extracellular LDH was determined after a two-hour period of treatment. A low level of LDH was found in cells kept under normoxic conditions whereas cells kept under nitrogen showed a significant release of LDH indicating severe cellular damage (Fig. 1b). If instead of nitrogen xenon was used to create such hypoxic condition, the LDH level remained at the same low level as in controls.

### Effect of the dopamine reuptake inhibitor GBR 1209

**Figure 2:**
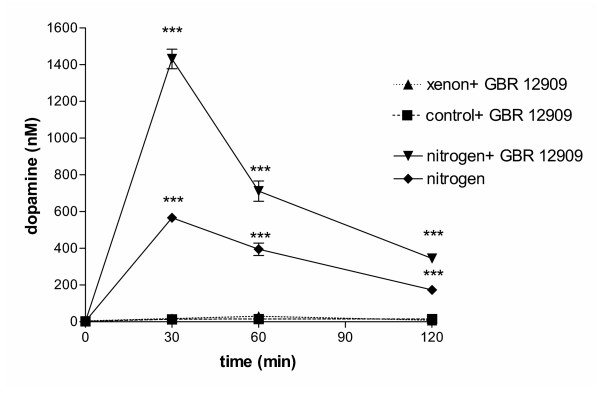
Effect of the dopamine uptake inhibitor GBR 1209 on dopamine release in hypoxic cells. If PC-12 cells were incubated in the presence of 5 nM of the dopamine uptake inhibitor GBR 1209, no effect was observed in cells incubated under normoxic conditions or in a xenon atmosphere. However, a much higher extracellular dopamine concentration was found in cells incubated in N_2 _and GBR 1209 than in N_2 _alone, indicating that indeed the release was intensified under hypoxic conditions. (n = 4; P < 0.0001 with respect to untreated controls, analyzed by 2-way ANOVA and Bonferroni posttests between GBR1209-treated and untreated groups).

Hypoxia-induced extracellular increase of dopamine could be caused either by elevated release of dopamine or by a reduced, or even inhibited, dopamine uptake. If hypoxia caused faster release but did not interfere with uptake, uptake-inhibitors would cause a higher concentration of dopamine in the extracellular space. On the other hand, if the release was constant but the re-uptake inhibited by hypoxia, additional inhibition of uptake by inhibitors would have no or little effect. In the presence of 5 nM of the dopamine reuptake inhibitor GBR 1209 the extracellular dopamine concentration did not change in a normoxic or xenon environment. However, in nitrogen the extracellular dopamine concentration did not reach exactly the same value as in pure nitrogen, the dopamine level was slightly but significantly reduced, thus supporting the view that hypoxia-induced extracellular dopamine increase was caused by an enhanced release of dopamine and – to a lesser extent – an interference with the uptake mechanism (Fig. 2).

### Effects of the dopamine receptor antagonists SCH 23390 and sulpiride

**Figure 3:**
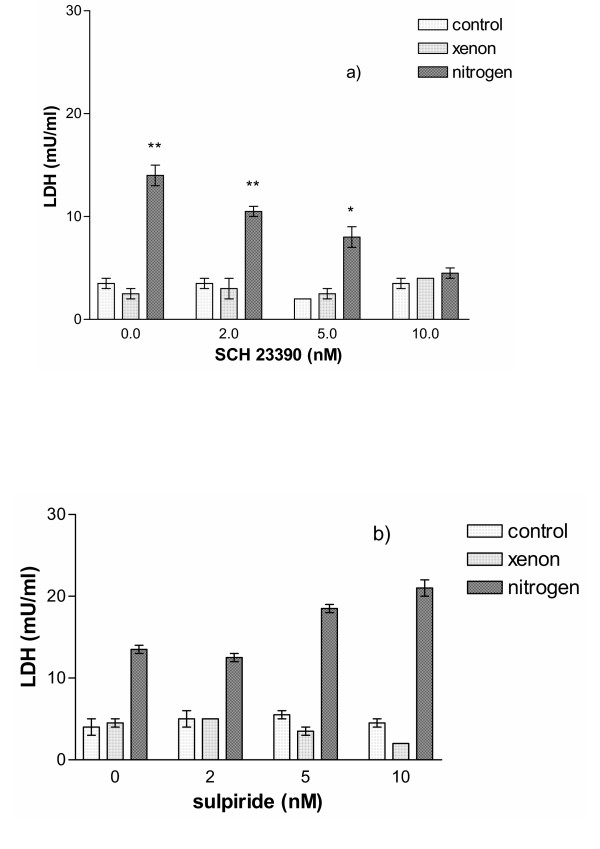
A. Effect of various concentrations of the D1-receptor antagonist SCH 23390 on cell survival after two-hour incubation. No effect was observed on PC-12 cells maintained under normal conditions or in xenon, however, for cells kept in nitrogen, a concentration-dependent decrease of cellular damage was found, indicating that the D1-receptor was involved to convey the cellular damage. (n = 4; **P < 0.01 and *P < 0.05 with respect to untreated controls). B. Effect of various concentrations of the D2-receptor antagonist sulpiride on cell survival after two-hour incubation. No protective effect of sulpiride was found for cells incubated in nitrogen, even a slight increase of cellular damage was seen with increasing concentrations of sulpiride.

To test if indeed the hypoxia-induced increase of extracellular dopamine itself caused the cell damage measured by the increase in extracellular LDH, dopamine receptor antagonists were used. Since they prevent dopamine binding they should provide protection of dopamine-induced damage. If the D1 receptor antagonist SCH 23390 was used during the incubation period, then at the highest dose of 10 nM, a reduction of nitrogen-induced external LDH-increase could be seen. However, even at this highest applied dose of SCH 23390, there was still only a less than 50% reduction in extracellular LDH (Fig. 3a). If, on the other hand, the D2 receptor antagonist sulpiride was used, no reduction in the nitrogen-induced LDH-release was found (Fig. 3b). Both compounds did not change the xenon-induced suppression of cellular damage.

### Cellular damage induced by external addition of dopamine

**Figure 4:**
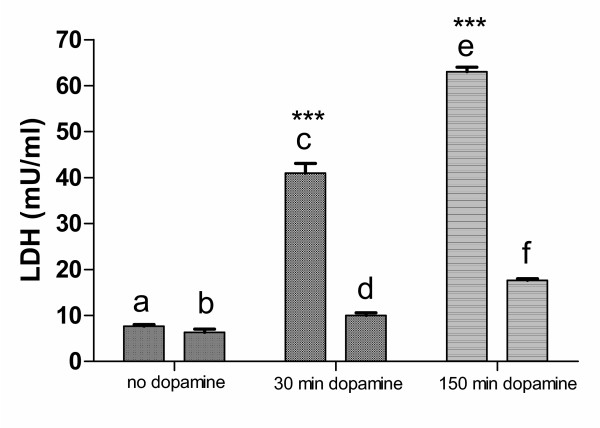
Dopamine-induced cellular damage. Cells were incubated for 150 min with and without 100 nM dopamine and LDH-release was determined. (a) untreated control; (b) untreated cells kept for 30 min in normoxia followed by 120 min in xenon-atmosphere; (c) cells under normoxic conditions treated for 30 min with 100 nM dopamine followed by 120 min in normal medium; (d) cells under normoxic conditions treated for 30 min with 100 nM dopamine, followed by 120 min incubation in xenon-atmosphere in normal medium without dopamine; (e) cells under normoxic conditions treated for 150 min with 100 nM dopamine; (f) cells under normoxic conditions treated for 30 min with 100 nM dopamine, followed by 120 min incubation in xenon-atmosphere in medium containing 100 nM dopamine. No significant difference was found between (a) and (b) whereas the difference between (c) and (d), and (e) and (f) was highly significant (P < 0.0001).

To analyze if indeed the increased external dopamine was detrimental to cells, they were incubated in the presence of 100 nM dopamine, either for 30 min followed by 120 min in normal medium, or continuously for 150 min. As shown in fig. 4, column (c), even the 30 min incubation with 100 nm dopamine (the lesser of the two dopamine challenges) was sufficient to cause considerable cell damage. Such damage was further increased if dopamine was present for the whole period of time of 150 min (column (e). We asked then if xenon not only prevented the release of dopamine in a hypoxic situation but could even reduce the damage caused by external dopamine. Cells were incubated for 30 min in normal buffer containing 100 nM dopamine followed by 120 min in xenon-saturated buffer, without dopamine. As shown in column (d), the dopamine-induced damage to the cells as seen in column (c) was significantly reduced. If the cells were incubated for 150 min in xenon-saturated buffer containing dopamine, even under those conditions the damage was low compared to cells exposed to dopamine in normal buffer (column (f)).

### Buffering of intracellular Ca^2+^-ions using BAPTA

**Figure 5:**
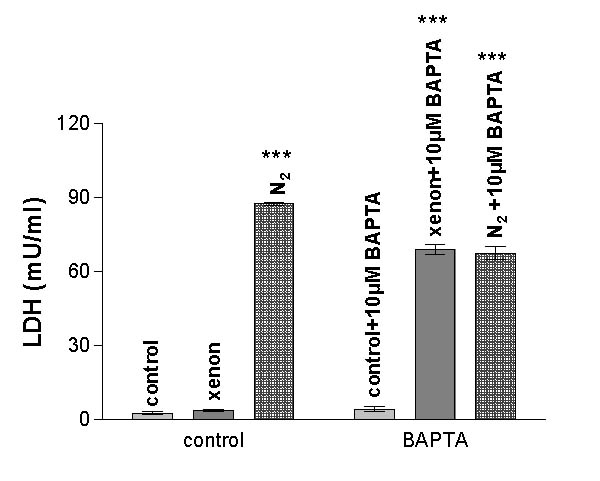
Effects of intracellular Ca^2+^-buffering by BAPTA. If PC-12 cells were preloaded with 10 μM BAPTA, the neuroprotective effect of xenon was strongly reduced. At the same time, however, a small reduction in cellular damage was observed in nitrogen-treated cells. (n = 5; P < 0.0001 analyzed by 2-way ANOVA and Bonferroni posttests between BAPTA-treated and untreated groups; no significant difference was found when the nitrogen-group was compared to the nitrogen-BAPTA group).

In order to test if changes in intracellular Ca^2+ ^were required for the neuroprotective effect of xenon, cells were incubated with the cell-permeant Ca-chelator BAPTA-AM. As shown in fig. 5, chelating intracellular Ca^2+ ^does not damage the cells *per se *(control + 10 μM BAPTA). Surprisingly, such chelation reduces significantly the neuroprotective effect of xenon, indicating an essential role for intracellular Ca^2+ ^for this effect to occur. A slight but significant reduction in cellular damage is observed when BAPTA-treated cells are incubated in nitrogen-saturated buffer.

### Comparison with another dopaminergic cell system

**Figure 6:**
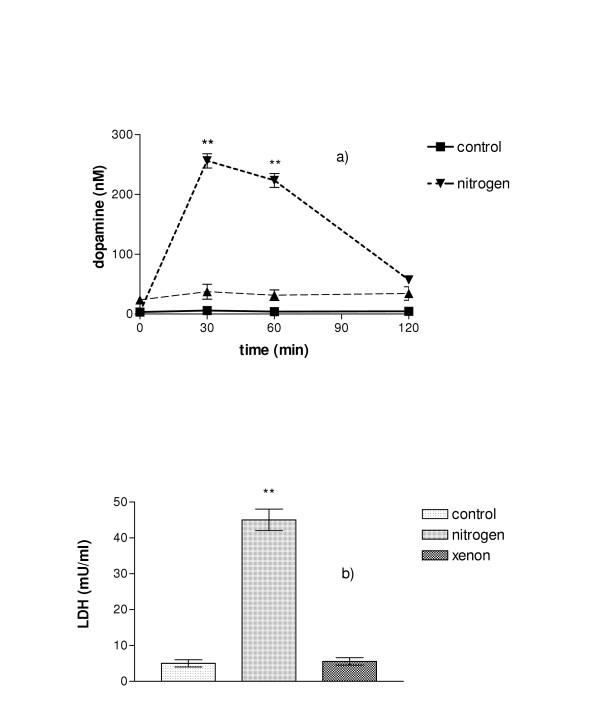
A. Dopamine release from mesencephalic dopaminergic cells under normoxic conditions, in N_2_, or in xenon. Mesencephalic cells containing dopaminergic neurons were exposed on day 14 after preparation for two hours either to normal atmosphere, or xenon or nitrogen. Dopamine was not released from cells maintained under normal conditions or in xenon whereas a significant amount of dopamine was liberated from cells maintained in nitrogen. B. Cellular damage in mesencephalic dopaminergic cells after two-hour incubation. If cells were kept in normal air, or in xenon, only a small amount of LDH was released. Much higher cellular damage was found when cells were incubated in nitrogen. (n = 4; **P < 0.01).

To exclude that the results obtained were limited to the PC-12-system itself, hypoxia-induced dopamine release and cell damage was investigated in rat embryonic primary mesencephalic cell cultures that are known to contain 0.5 – 1.5% of dopaminergic cells [26]. As shown in fig. 6, in an hypoxic atmosphere a very similar pattern of dopamine and LDH release is obtained compared to PC-12 cells. Xenon prevents also in these primary cells the hypoxia-induced neurotransmitter and LDH release.

## Discussion

In hypoxia/ischemia a key feature of secondary damage after the primary neuron-damaging event is the over-release of neurotransmitters [17]. Consequently, an interference with the hypoxia-induced release mechanism with respect to its control systems may be extremely useful to reduce cellular damage. The results presented here show that xenon has such properties, namely to prevent cellular damage and neurotransmitter release in a hypoxic situation thus qualifying it as an almost ideal early neuroprotectant. Concerning possible cellular targets for xenon, a first indication for the participation of Ca^2+^-regulated events was obtained when it was shown that xenon blocked cells in metaphase and that the block could be lifted by artificial small intracellular Ca^2+^- increases [18]. Since the CaM KII complex is known to play a decisive role in the metaphase/anaphase transition, it was tested if the CaMKII-inhibitor KN-93 had likewise metaphase-blocking properties. Such effects were obtained [19]. It is well known that in dopaminergic differentiated PC-12 cells, the CaMKII complex is involved in the regulation of neurotransmitter release [20-22] as well as its participation in a multitude of other Ca^2+^-dependent regulatory events [23]. Thus, it appears to be plausible that one of the targets for xenon might be the CaMKII complex, either directly or via interference with other Ca^2+^-dependent systems. One of those may be the Ca^2+^/calmodulin-activated calcineurin system that has been implicated in the regulation of monoamine release [24]. Alternatively, xenon might interact upstream of these regulatory systems with other Ca^2+^-dependent events required to occur in hypoxia-induced cell damage. Such a scenario is suggested by our demonstration that the neuroprotective effect of xenon is strongly reduced if PC-12 cells are loaded with BAPTA. Thus, at present all evidence obtained by us [13,14,18,19] and others [15,16] establish a complex and composite picture of targets susceptible to xenon including NMDA receptors, Ca^2+^-regulating and -regulated systems up to the activaton of transcription factors whereby such targets are probably not essentially and sequentially linked to each other.

To summarize briefly our main findings: (1) The presence of xenon blocks hypoxia-induced dopamine release in dopaminergic cells. (2) Hypoxia-induced dopamine increase is caused by an enhanced release of dopamine rather than a reduced uptake of dopamine. (3) When measuring LDH release as a marker of cellular damage, xenon was found to block such release, which suggests that xenon reduces hypoxia-induced cellular damage. (4) Increased extracellular dopamine can damage dopaminergic cells directly. This is mainly mediated by D1 receptor agonism rather than D2. (5) Such direct extracellular dopamine-induced damage can be reduced by the presence of xenon, even when the increase in extracellular dopamine has not been caused by an episode of cellular hypoxia. (6) The above described protective effects of xenon depend on the presence of calcium ions.

Further studies will show if indeed in the hypoxic cell multiple intracellular targets exist for xenon and how they are orchestrated together to result in cellular protection.

## Conclusions

Based on the present results obtained with NGF-differentiated PC-12 cells and on the literature cited in this paper, xenon appears to be a neuroprotectant for a broad spectrum of neuronal cells; given its proven non-toxicity based on its long clinical use, it may come close to fulfilling the requirements for an ideal or "gold standard" neuroprotectant.

## Methods

### Cells

Rat pheochromocytoma cells (PC-12) were maintained in RPMI 1640 medium containing 5% fetal calf serum, 10% horse serum, at 37°C, 5% CO_2_. For experiments, cells were seeded in 24-well plates at a density of 1 × 10^5 ^cells/well and nerve-growth-factor (Promega, Heidelberg, Germany) was added (0.4 μg/ml) whereupon cells entered differentiation. They were used on day five after the addition of growth factor [25]. Primary dopaminergic cells from rat embryonic brain were prepared as described (26) and used on day 14.

### Determination of dopamine and LDH

Hypoxia-treatment was performed as described [13]. Samples from individual wells were taken at the intervals indicated and deproteinated using 5% perchloric acid (1:1 = vol/vol). Supernatants were transferred to Eppendorff tubes and the same volume (0.5 ml) of 0.4 M perchloric acid was added, mixed on vortex and centrifuged (6000 rpm, 3 min) to remove cell debris. Dopamine concentration was determined by high-pressure liquid chromatography (Bio-Tek, Neufahrn, Germany) using an electrochemical detector (Biometra, Göttingen, Germany). Cellular damage after the experiment was assessed by measuring spectrophotometrically the concentration of LDH in the original supernatant, before the addition of perchloric acid (Roche Diagnostics, Mannheim, Germany).

### Chemicals

Gases were supplied by AGA-Linde (Berlin, Germany). 1,2-*bis*(2-Aminophenoxy)ethane-N,N,N',N'-tetraacetic acid *tetrakis*(acetoxymethyl) ester (BAPTA-AM) was purchased from Molecular Probes, (Leiden, The Netherlands), and all standard chemical products were obtained from Merck (Berlin, Germany).

### Statistical analysis

All experiments were repeated at least five times, i.e. in five different plates on five different days. The data were presented as means ± SEM. The results of multiple groups were analyzed using one-way ANOVA with Dunnett's multiple comparison post test or two-way ANOVA with Bonferroni posttests using GraphPad Prism version 3.00 for Windows, GraphPad Software, San Diego California USA. Differences with *p *values less than 0.05 were considered statistically significant.

## Authors' contributions

CP conceived the study, participated in its design and coordination, carried out the cellular studies involving the various gas treatments, participated in the preparation of the cells, and drafted the manuscript. PB and WS participated in the design of the experiments and the gas applications, JM performed neurotransmitter analysis and LDH determinations, and WK participated in the design of the study and performed the statistical analysis. All authors read and approved the final manuscript.
